# MicroRNA-325-3p prevents sevoflurane-induced learning and memory impairment by inhibiting Nupr1 and C/EBPβ/IGFBP5 signaling in rats

**DOI:** 10.18632/aging.102942

**Published:** 2020-03-19

**Authors:** Lili Xu, Qi Xu, Fang Xu, Wenxin Zhang, Qing Chen, Hui Wu, Xinzhong Chen

**Affiliations:** 1 Department of Anesthesiology, Women’s Hospital, Zhejiang University School of Medicine, Hangzhou, Zhejiang Province, China

**Keywords:** sevoflurane, miR-325-3p, Nupr1, C/EBPβ, IGFBP5

## Abstract

Endoplasmic reticulum stress-induced neuronal apoptosis contributes to neurotoxicity observed after sevoflurane exposure. However, the molecular mechanism underlying the resulting learning and memory impairments remains unknown. Here, we investigated the roles of miR-325-3p and Nupr1 in sevoflurane-induced learning and memory impairments in neonatal rats and HCN-2 human cortical neuronal cells. We found that in both neonatal rats and HCN-2 cells, sevoflurane exposure impairs learning and memory in neonatal rats and increases expression of Nupr1, the endoplasmic reticulum stress marker proteins C/EBPβ and IGFBP5, and the apoptosis-related protein markers cleaved-Caspase-3 and Bax. Using bioinformatics tools to identify microRNAs that bind to Nupr1, we found that miR-325-3p is downregulated in hippocampal neurons exposed to sevoflurane. Moreover, Nupr1 knockdown and miR-325-3p overexpression improved the rats’ performance in learning and memory tests and reduced sevoflurane-induced apoptosis *in vitro* and *in vivo*. These results suggest that miR-325-3p blocks sevoflurane-induced learning and memory impairments by inhibiting Nupr1 and the downstream C/EBPβ/IGFBP5 signaling axis in neonatal rats. MiR-325-3p may therefore be a useful therapeutic target in sevoflurane-induced neurotoxicity.

## INTRODUCTION

Exposure to sevoflurane during brain development can cause long-term learning and memory deficits, long-lasting complex social and emotional behavior disorders, and even increase the risk of Alzheimer's disease [1]. The degree of damage to synaptic function in developing cortical and hippocampal neurons differs depending on brain region, length of exposure, and age [2]. Accumulating preclinical evidence suggests that the mechanisms underlying sevoflurane-induced neurotoxicity include neuronal dysregulation, disorders of neurogenesis and hippocampal and cortical development, increases in neuron and oligodendrocyte death, and decreased nerve growth/nutrient factor expression in rodents and nonhuman primates [2, 3]. However, the specific molecular mechanisms remain unknown.

Sevoflurane exposure can increase the expression of endoplasmic reticulum (ER) stress markers such as CCAAT/enhancer-binding protein homologous (C/EBPβ) and Caspase-3 both *in* *vitro* and *in* *vivo*, suggesting that ER stress response might play an important role in sevoflurane-induced neurotoxicity [4, 5]. Both IRE1 and PERK are crucial for the detection of and injury induced by ER stress [6]. After activation by GRP78, they activate downstream signaling pathways during the initiation of ER stress, resulting in decreased protein translation and increased chaperone production that help restore ER homeostasis [6]. Specifically, PERK and IRE1 activate the ATF4 and ASK1 signaling pathways, respectively, which increase C/EBPβ and JNK expression during prolonged or severe ER stress. Continuous upregulation of C/EBPβ and JNK can promote cell death [7].

As a member of the high mobility group of transcriptional regulators, Nuclear protein 1 (Nupr1) mediates cellular stress and metastasis [8]. Despite its small size and relatively simple structure, Nupr1 functions in several genetic and biochemical signaling pathways [8]. Nupr1 expression is low under normal physiological conditions, but can be induced by and plays an important role in hypoxia, oxidative stress, and DNA damage [8]. A recent report found that Nupr1 is involved in methamphetamine (METH)-induced neuronal apoptosis and autophagy through the ER stress signaling pathway [9]. Santofimia et al. [10] demonstrated that inactivation of Nupr1 in pancreatic cancer cells resulted in ER stress that induced mitochondrial malfunction, decreased ATP production, and ultimately promoted cell death via programmed necrosis. In previous bioinformatics analyses performed to identify microRNAs that bind to Nupr-1, we found that microRNA-325-3p (miR-325-3p) was downregulated in neurons exposed to sevoflurane. Here, we investigated the effects and molecular mechanisms of Mir-325-3p and its target gene Nupr1 on sevoflurane-induced neurotoxicity in neonatal rats and the HCN-2 human neuronal cell line.

## RESULTS

### Sevoflurane did not alter arterial blood pressure or arterial blood gas levels

To assess its effects on the developing brain, we exposed rats to air with or without 3.4% sevoflurane for six hours. There were no signs of cardiorespiratory dysfunction after sevoflurane administration, and SaO_2_, PaO_2_, PaCO_2_, pH, and MAP did not differ between the two groups. (Table 1). These results suggest that sevoflurane exposure had no detrimental physiological effects.

**Table 1:** Arterial blood pressure and arterial blood gas analysis.

	**Time**	**pH**	**PaCO_2_, mmHg**	**PaO_2_, mmHg**	**SaO_2_ %**	**MAP, mmHg**
Air (n = 6)	0 h	7.41±0.02	41.1±4.6	98.7±7.4	99.4±0.5	51±6
	6 h	7.40±0.03	42.9±3.8	99.6±8.3	99.7±0.6	49±4
3.4% SEV (n = 6)	0 h	7.42±0.06	42.6±5.4	98.5±6.4	98.3±0.3	50±5
	6 h	7.39±0.04	43.5±4.7	99.4±8.9	99.4±0.5	47±3

Neonatal sevoflurane exposure did not cause significant cardiorespiratory dysfunction. Arterial blood pressure and arterial blood gas analysis showed no significant differences between rats exposed to 3.4% sevoflurane exposure for six hours and controls. p>0.05. N=6. PaCO_2_=arterial carbon dioxide tension; PaO_2_=arterial oxygen tension; SaO_2_=arterial oxygen saturation.

### Sevoflurane impaired learning and memory and increased neural apoptosis and Nupr1 mRNA levels in neonatal rats

**Figure 1:**
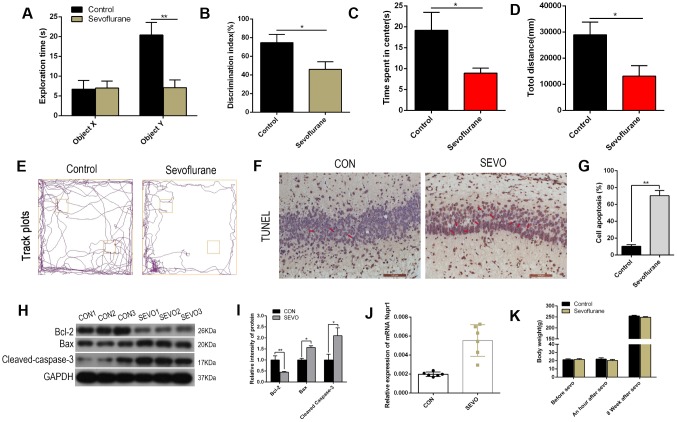
**Sevoflurane impaired learning and memory, induced neuronal apoptosis, and increased Nupr1 mRNA expression in neonatal rats.** Neonatal rats were separated into two groups of 12 each. The control group was exposed to air, while the sevoflurane (SEVO) group was exposed to 3.4% SEVO, for 6 hours. (**A**, **B**) A novel object recognition test was conducted 8 weeks after SEVO exposure. (**A**) Exploration times during the recognition session for the familiar (X) and novel (Y) objects. (**B**) The discrimination index indicates time spent exploring the novel object relative to total exploration time for both the novel and familiar objects. (**C**–**E**) An open field test was conducted 8 weeks after SEVO exposure. (**C**) Time spent in the center of the open field during the 5 min exploration period. (**D**) Total distance traveled during the 5 min exploration period. (**E**) Traces showing rats’ movements during the 5 min exploration period. (**F**–**G**) TUNEL staining in the rat hippocampus; representative images (**F**) and quantification (**G**). (**H**–**I**) Western blotting for Cleaved-Caspase-3, Bax, and Bcl-2 in rat brain; representative images (**H**) and quantification (**I**). (**J**) RT-qPCR for Nupr1 mRNA. (**K**) Rat body weights at seven days after birth and eight weeks after sevoflurane or air exposure. **p<0.05. N=6.

Neonatal rats in the control group were exposed to air, while the sevoflurane group was exposed to 3.4% sevoflurane, for six hours. In a novel object recognition test, neonatal rats exposed to sevoflurane exhibited shorter exploration times for the novel object (object Y) during the recognition session and a lower discrimination index compared to the control group; the two groups did not differ in time spent exploring the familiar object (object X) (Figure 1A, 1B). The rats were also assessed in an open field test eight weeks after sevoflurane exposure. Neonatal rats exposed to sevoflurane spent less time in the center of the open field and traveled shorter total distances during the five-minute exploration period compared to the control group (Figure 1C–1E). These results suggest that sevoflurane impaired both learning and memory in neonatal rats. In addition, numbers of TUNEL-positive neurons in the rat hippocampus increased after sevoflurane exposure (representative images: Figure 1F; quantification: Figure 1G). Western blots revealed that levels of the apoptotic cell markers Cleaved-Caspase-3 and Bax increased, while Bcl-2 levels decreased, in the rat hippocampus after sevoflurane exposure (representative images: Figure 1H; quantification: Figure 1I). Nuclear protein 1 (Nupr1/com1/p8) is a member of the high mobility group of transcriptional regulators and is involved in neuronal apoptosis and autophagy through the ER stress signaling pathway. Nupr1 mRNA levels increased in the rat brain after sevoflurane exposure (Figure 1J). Finally, body weights did not differ between the groups at either seven days after birth or eight weeks after sevoflurane or air exposure (Figure 1K). Together, these data suggest that sevoflurane impairs learning and memory and increases neural apoptosis and Nupr1 mRNA levels in neonatal rats.

### Sevoflurane increased Nupr1, C/EBPβ, and IGFBP5 expression and induced apoptosis in HCN-2 neuronal cells

**Figure 2:**
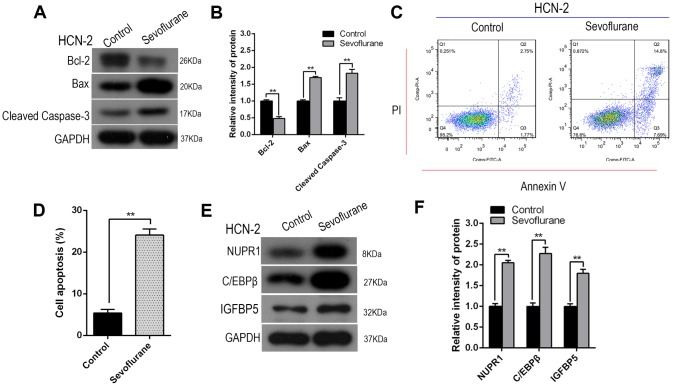
**Sevoflurane increased Nupr1, C/EBPβ, and IGFBP5 expression and induced apoptosis in HCN-2 neuronal cells.** Identical numbers of HCN-2 neuronal cells were exposed to fresh gas (21% O_2_, 5% CO_2_, remainder N_2_) alone or with the addition of 3.4% sevoflurane for 6 h and then subjected to analysis. (**A**, **B**) Western blotting for Cleaved-Caspase-3, Bax, and Bcl-2 in 3.4% SEVO-exposed vs. control HCN-2 cells; representative images (**A**) and quantification (**B**). (**C**, **D**) Annexin V-FITC/PI staining and flow cytometry analysis; representative images (**C**) and quantification (**D**). (**E**, **F**) Western blotting for Nupr1, C/EBPβ, and IGFBP5 in 3.4% SEVO-exposed vs. control HCN-2 cells; representative images (**E**) and quantification (**F**). **p<0.05. N=6.

Western blot analysis revealed that Cleaved-Caspase-3 and Bax levels increased, while Bcl-2 levels decreased, after sevoflurane exposure in HCN-2 cells (representative images: Figure 2A; quantification: Figure 2B). Annexin V-FITC/PI staining and flow cytometry analysis also showed that apoptosis rates, including both early and late apoptosis, increased in HCN-2 cells after sevoflurane exposure (Figure 2C, 2D).

The transcription factor C/EBPβ is an important regulator of cell apoptosis and autophagy. Insulin-like growth factor binding protein (IGFBP5) is a proapoptotic factor that mediates Meth-induced neuronal apoptosis. Western blot analysis demonstrated that Nupr1, C/EBPβ, and IGFBP5 levels all increased after sevoflurane exposure in HCN-2 neuronal cells (representative images: Figure 2E; quantification: Figure 2F). Collectively, these results suggested that increased Nupr1, C/EBPβ, and IGFBP5 protein expression may contribute to sevoflurane-induced neuronal apoptosis.

### Nupr1 knockdown attenuated sevoflurane-induced apoptosis and decreased C/EBPβ and IGFBP5 expression in HCN-2 neuronal cells

**Figure 3:**
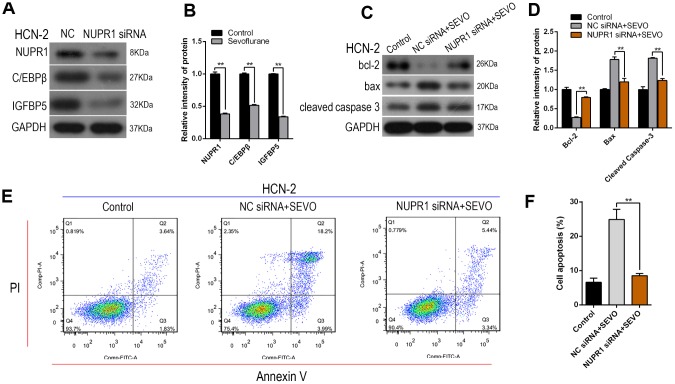
**Nupr1 knockdown attenuated sevoflurane-induced apoptosis and decreased C/EBPβ and IGFBP5 expression in HCN-2 neuronal cells.** HCN-2 neuronal cells were transfected with NC or Nupr1 siRNA and then exposed to fresh gas (21% O_2_, 5% CO_2_, remainder N_2_) alone or with the addition of 3.4% sevoflurane for 6 h. (**A**, **B**) Western blotting for Nupr1, C/EBPβ, and IGFBP5 in 3.4% SEVO-exposed vs. control HCN-2 cells; representative images (**A**) and quantification (**B**). (**C**, **D**) Western blotting for Cleaved-Caspase-3, Bax, and Bcl-2 in 3.4% SEVO-exposed vs. control HCN-2 cells; representative images (C) and quantification (**D**). (**E**, **F**) Annexin V-FITC/PI staining and flow cytometry analysis; representative images (**E**) and quantification (**F**). **p<0.05. N=6.

Next, we examined whether Nupr1 is involved in sevoflurane-induced ER stress as indicated by increased C/EBPβ and IGFBP5 expression. Sevoflurane exposure again increased C/EBPβ and IGFBP5 protein expression in HCN-2 cells compared to the control group, and simultaneous administration of Nupr1 siRNA attenuated this increase (Figure 3A, 3B). These results suggest that Nupr1 may be an upstream regulator of C/EBPβ and IGFBP5, which play critical roles in sevoflurane-induced ER stress.

We then investigated whether Nupr1 knockdown inhibited sevoflurane-induced apoptosis in HCN-2 neuronal cells using Western blot and flow cytometry. Sevoflurane exposure increased the expression of several proteins, including Nupr1 and cell apoptosis markers Cleaved-Caspase-3 and Bax, but decreased Bcl-2 levels. Notably, expression of these proteins did not differ from control group levels after co-administration of sevoflurane and Nupr1 siRNA (Figure 3C, 3D). Annexin V-FITC/PI staining and flow cytometry analysis showed that apoptosis rates, including both early and late apoptosis, decreased after co-exposure to sevoflurane and Nupr1 siRNA relative to sevoflurane alone (Figure 3E, 3F). These results suggest that knockdown of Nupr1 expression can inhibit sevoflurane-induced apoptosis in HCN-2 neuronal cells.

### Sevoflurane downregulated Mir-325-3p in HCN-2 neuronal cells

**Figure 4:**
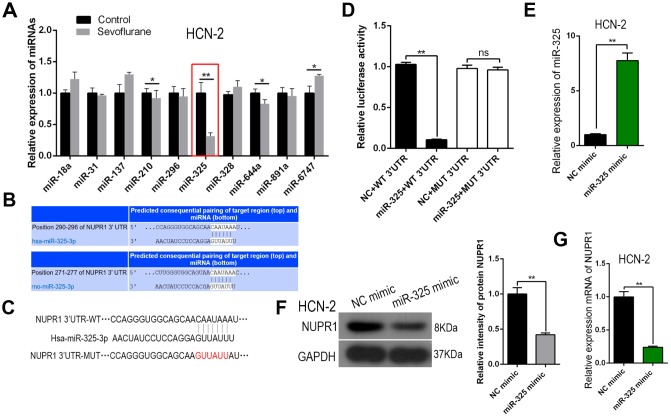
**miR-325-3p, which suppressed Nupr1 translation, was downregulated by sevoflurane in neuronal cells.** (**A**) RT-qPCR for 10 Nupr1-targeting miRNAs in HCN-2 cells exposed to fresh gas (21% O_2_, 5% CO_2_, remainder N_2_) alone or with the addition of 3.4% sevoflurane for 6 h. (**B**, **C**) Bioinformatics analysis showing predicted binding of miR-325-3p to the 3'-UTR of rat (**B**) and human (**C**) Nupr1 mRNA. (**D**) Wild-type Nupr1 mRNA (Nupr1 3'-UTR) and Nupr1 mRNA with a mutation in the 3'-UTR miR-325-3p-binding site (Nupr1 3'-UTR mut) were prepared. A dual-luciferase reporter assay was performed using all combinations of miR-325-3p-modifying and Nupr1 3'-UTR plasmids. (**E**) MiR-325-3p was overexpressed using an miR-325-3p mimic in HCN-2 neuronal cells. Controls were transfected with NC mimic. miR-325-3p levels in these cells were measured using RT-qPCR. (**F**, **G**) Western blot (**F**) and RT-qPCR (**G**) for Nupr1 levels in miR-325-3p-modified HCN-2 cells. **p<0.05. N=6.

We hypothesized that sevoflurane might activate Nupr1 by suppressing an miRNA that targets Nupr1. We therefore performed an miRNA assay on HCN-2 cells exposed to control gas or 3.4% sevoflurane for six hours and identified miR-325, a Nupr1-binding miRNA that was downregulated by sevoflurane (Figure 4A). Moreover, miR-325 was predicted to bind to the Nupr1 mRNA 3′-UTR in both rats (Figure 4B) and humans (Figure 4C).

### Mir-325-3p suppressed translation of Nupr1 in HCN-2 neuronal cells

Plasmids containing Nupr1 mRNA with the wild-type 3′-UTR sequence (WT-Nupr1 3′-UTR) or Nupr1 mRNA with a mutation in the 3′-UTR at the miR-325-3p-binding site (MUT-Nupr1 3′-UTR) were prepared and used in combination with plasmids that modified miR-325-3p expression in a dual-luciferase reporter assay. The results showed that binding of miR-325-3p to the Nupr1 mRNA 3′-UTR inhibited Nupr1 protein translation in HCN-2 cells (Figure 4D). Next, changes in Nupr1 expression were assessed when miR-325-3p was overexpressed via transfection of an miR-325 mimic plasmid in HCN-2 cells; an NC mimic plasmid served as a control. qRT-PCR confirmed that the overexpression plasmid successfully increased miR-325-3p levels in these cells (Figure 4E). Additionally, Nupr1 protein and mRNA levels both decreased when miR-325-3p was overexpressed (Figure 4F, 4G). Together, these data suggest that miR-325-3p targets Nupr1 to inhibit its translation in neurons.

### Overexpression of Mir-325-3p alleviated sevoflurane-induced apoptosis in HCN-2 neuronal cells

**Figure 5:**
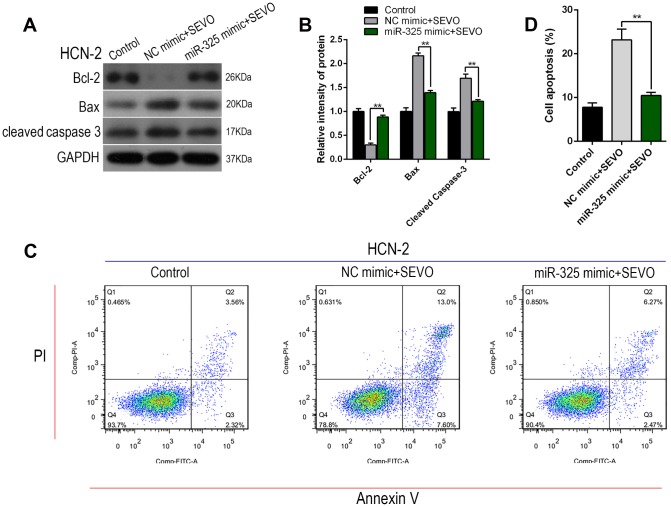
**Overexpression of Mir-325-3p inhibited sevoflurane-induced apoptosis in HCN-2 neuronal cells.** HCN-2 neuronal cells were transfected with miR-325 mimic or NC mimic and then exposed to fresh gas (21% O_2_, 5% CO_2_, remainder N_2_) alone or with the addition of 3.4% sevoflurane for 6 h. (**A**, **B**) Western blotting for Cleaved-Caspase-3, Bax, and Bcl-2 in 3.4% SEVO-exposed vs. control HCN-2 cells; representative images (**A**) and quantification (**B**). (**C**, **D**) Annexin V-FITC/PI staining and flow cytometry analysis; representative images (**C**) and quantification (**D**). **p<0.05. N=6.

Next, we explored whether miR-325 overexpression inhibited sevoflurane-induced apoptosis in HCN-2 neuronal cells using Western blot and flow cytometry. Sevoflurane exposure together with NC mimic transfection increased the expression of Nupr1 and cell apoptosis markers Cleaved-Caspase-3 and Bax, but decreased Bcl-2 expression. Notably, co-exposure to sevoflurane and miR-325 mimic plasmid reversed these expression changes to sevoflurane group levels (Figure 5A, 5B). Annexin V-FITC/PI staining and flow cytometry analysis were also performed to confirm the effects of miR-325 expression on sevoflurane-induced apoptosis. Co-exposure to sevoflurane and miR-325 mimic decreased apoptosis rates, including both early and late apoptosis (Figure 5C, 5D). These results indicated that miR-325 attenuated sevoflurane-induced neuronal apoptosis.

### Upregulation of Mir-325-3p expression attenuated sevoflurane-induced learning and memory impairment in neonatal rats

**Figure 6:**
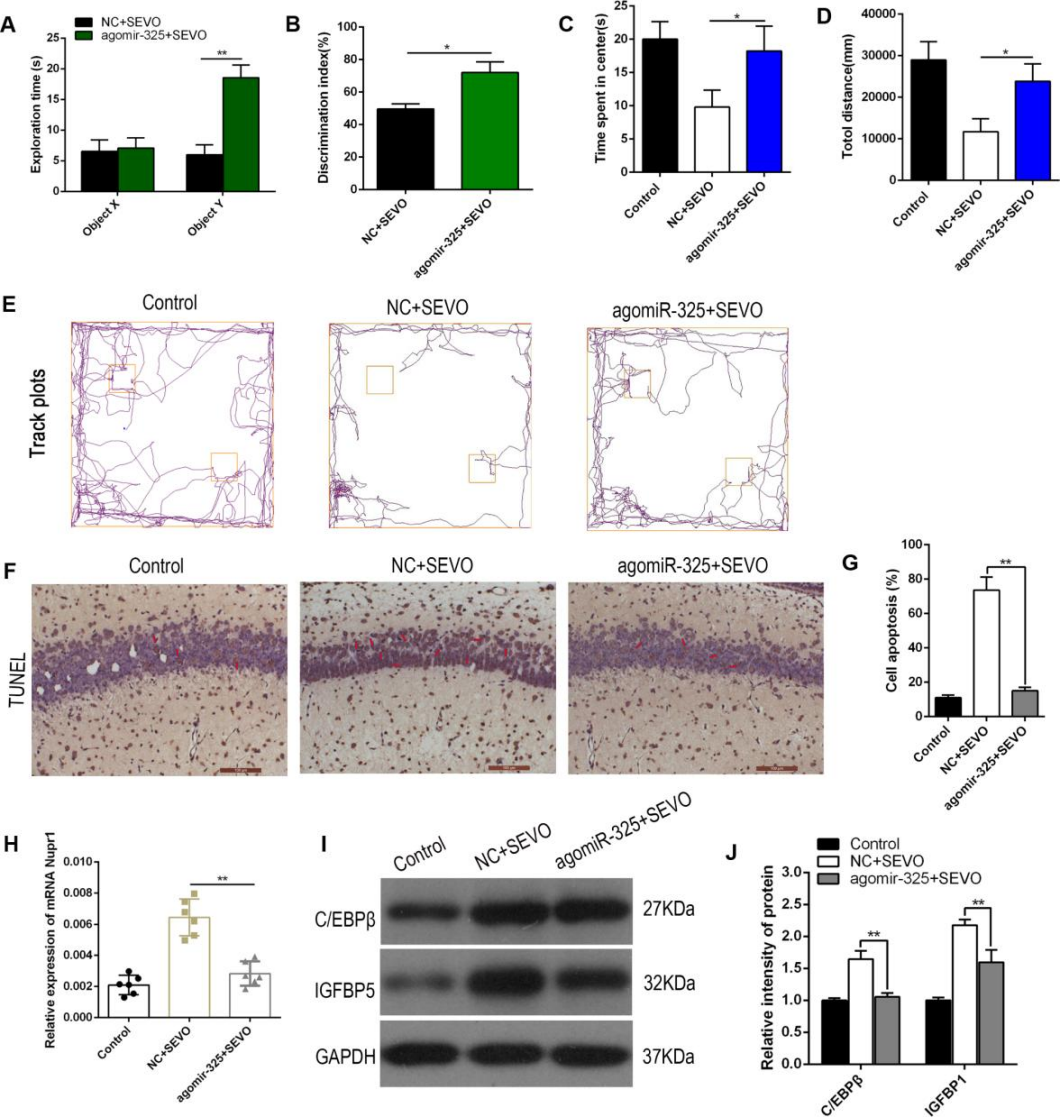
**Upregulation of Mir-325-3p expression attenuated sevoflurane-induced learning and memory impairments in neonatal rats.** AgomiR-325-3p or agomiR-NC was intracranially injected bilaterally into the hippocampi of neonatal rats (n=6 per group). One day after injection, the rats were exposed to SEVO. (**A**, **B**) A novel object recognition test was performed 8 weeks after SEVO exposure. (**A**) Exploration times during the recognition session for familiar (X) and novel (Y) objects. (**B**) The discrimination index indicates time spent exploring the novel object relative to total exploration time for both the novel and familiar objects. (**C**–**E**) An open field test was performed 8 weeks after SEVO exposure. (**C**) Time spent in the center of the open field during the 5 min exploration period. (**D**) Total distance traveled during the 5 min exploration period. (**E**) Traces showing rats’ movements during the 5 min exploration period. (**F**, **G**) TUNEL staining in the rat hippocampus; representative images (**F**) and quantification (**G**). (**H**) RT-qPCR for Nupr1 mRNA. (**I**–**J**) Western blotting for C/EBPβ and IGFBP5 in the rat hippocampus; representative images (**I**) and quantification (**J**). **p<0.05. N=6.

To evaluate the effects of miR-325-3p upregulation on learning and memory in rats after sevoflurane exposure, agomiR-325-3p was intracranially injected bilaterally into the hippocampi of neonatal rats (5 nmol per side every two days, 4 injections total). One day after the final injection, the rats were exposed to sevoflurane. In the novel object recognition test, neonatal rats that had received agomiR-325-3p prior to sevoflurane exposure spent more time exploring the novel object (object Y) during the recognition session and had higher discrimination index values than rats that had received agomiR-NC; there were no differences between the groups in exploration time for the familiar object (object X) (Figure 6A, 6B). Moreover, neonatal rats that had received agomiR-325-3p spent more time in the center of the open field and traveled longer total distances during the five-minute open field test compared to rats that had received agomiR-NC (Figure 6C–6E). In addition, numbers of TUNEL-positive neurons decreased in hippocampi of rats co-exposed to sevoflurane and agomiR-325-3p relative to those exposed to sevoflurane alone (representative images: Figure 6F; quantification: Figure 6G). Nupr1 mRNA levels also decreased in the brains of rats co-exposed to sevoflurane and agomiR-325-3p compared to those exposed to sevoflurane alone (Figure 6H). Notably, we explored whether miR-325 upregulation inhibited sevoflurane-induced ER stress as indicated by increased C/EBPβ and IGFBP5 expression in the rat hippocampus using Western blot. Sevoflurane exposure together with agomiR-NC increased the expression of C/EBPβ and IGFBP expression. Importantly, co-exposure to sevoflurane and agomiR-325-3p reversed these expression changes to sevoflurane group levels (Figure 6I, 6J). Together, these data suggest that miR-325-3p overexpression and the associated decrease in Nupr1 mRNA levels blocked sevoflurane-induced deficits in learning and memory in neonatal rats.

## DISCUSSION

In the present study, we found that sevoflurane exposure impaired neonatal rats’ performance in behavioral learning and memory tests, increased the expression of Nupr1 and the ER stress protein markers C/EBPβ and IGFBP5, and also promoted apoptosis in rat hippocampal neurons and HCN-2 neuronal cells. Nupr1 knockdown and miR-325-3p overexpression both significantly improved rats’ performance in the novel object recognition and open field tests and reduced sevoflurane-induced apoptosis *in* *vitro* and *in* *vivo*. These results suggest that miR-325-3p plays a vital role in sevoflurane-induced neurotoxicity not only in neonatal rats but also in cultured neuronal cells.

In previous studies [11–14], we reported that sevoflurane exposure can induce neuronal necrosis and apoptosis *in* *vitro* and in various brain regions, including cortex and hippocampus. However, the mechanism underlying these effects was unknown. A growing body of research has demonstrated that ER stress is involved in apoptosis and autophagy that contribute to neuronal degeneration after sevoflurane exposure. Zhou et al. [15] demonstrated that 4.1% sevoflurane treatment for six hours induced ER stress, which antagonizes sevoflurane-induced apoptosis in H4 human neuroglioma cells. Moreover, Shen et al. [16] found that repeated sevoflurane exposure upregulated proteins related to ER stress in the hippocampus of young rats, while the ER stress inhibitor tauroursodeoxycholic acid reversed sevoflurane-induced changes in levels of synaptic plasticity proteins. Liu et al. [7]. showed that inhibition of protein tyrosine phosphatase 1B, an ER membrane protein that activates ER stress, mitigated sevoflurane-induced neurodegeneration in the developing brain and eventually improved cognitive function. In line with these studies, we found that sevoflurane impaired learning and memory in novel object recognition and open field tests, induced neuronal apoptosis, and upregulated Nupr1 mRNA levels in neonatal rats. Similarly, sevoflurane treatment caused neuronal apoptosis and increased Nupr1, C/EBPβ, and IGFBP5 protein expression in HCN-2 neuronal cells. These results suggest that ER stress contributed to sevoflurane-induced neuronal apoptosis and learning and memory deficits, and that inhibiting ER stress response during sevoflurane anesthesia may help prevent these adverse effects.

Stress induces expression of the Nupr1 gene, which functions in several biochemical pathways and is involved in autophagy-dependent cell survival and apoptosis- and necrosis-induced cell death. Matsunaga et al. [17] demonstrated that Nupr1 knockdown reduced cell proliferation and increased apoptosis, suggesting that Nupr1 promotes cell survival and cytoprotective autophagy. In agreement with those results, Santofimia et al. [10] found that Nupr1 downregulation induced mitochondrial failure characterized by loss of mitochondrial membrane potential, a strong increase in ROS production, and concomitant relocalization of mitochondria to the vicinity of ER. Furthermore, expression of some ER stress response-associated genes decreased in Nupr1-deficient cells. Collectively, this evidence indicates that inactivation of Nupr1 promotes ER stress-induced mitochondrial malfunction, deficient ATP production, and ultimately cell death mediated by programmed necrosis. Xu et al. [9] showed that methamphetamine (Meth) exposure increased expression of Nupr1 and the ER stress protein markers C/EBPβ and Trib3, and also activated apoptosis and autophagy in rat primary neurons. Furthermore, silencing Nupr1 expression partly alleviated Meth-induced apoptosis and autophagy *in* *vitro* and *in* *vivo*. Here, we found that Nupr1 knockdown reduced sevoflurane-induced apoptosis and decreased C/EBPβ and IGFBP5 protein expression in neuronal cells. Our results confirm that Nupr1 protein not only regulates ER stress response, but also promotes sevoflurane-induced apoptosis through the C/EBPβ and IGFBP5 pathway in neuronal cells.

Recently, miR-325-3p has been implicated in the progression of several kinds of carcinoma and in organ dysfunction. Zhang et al. [18] showed that miR-325-3p overexpression attenuated the severity of cardiac tissue injury, decreased infarct sizes, and effectively ameliorated RIPK1/RIPK3/p-MLKL axis-induced necroptosis during myocardial infarction (MI). In addition, Yan et al. [19] found that miR-325-3p attenuated secondary injury after spinal cord injury (SCI) by inhibiting the EGFR/MAPK signaling pathway, microglial activation, and the release of inflammatory cytokines, suggesting that miR-325-3p might be a useful therapeutic target for SCI. Here, overexpression of miR-325-3p alleviated sevoflurane-induced apoptosis in HCN-2 neuronal cells and attenuated sevoflurane-induced learning and memory impairments in neonatal rats, highlighting the crucial role of miR-325-3p in sevoflurane-induced hippocampal neurotoxicity.

In summary, this study provides the first evidence that miR-325-3p inhibits sevoflurane-induced apoptosis by targeting Nupr1 and the downstream C/EBPb/IGFBP5 signaling pathway in both rat and human neuronal cells. MiR-325-3p might therefore be a therapeutic target in sevoflurane-induced neurotoxicity that can help prevent sevoflurane-induced learning and memory deficits in rats. Further studies of the specific mechanisms of ER stress during sevoflurane-induced apoptosis would improve our understanding of the functional roles of miR-325-3p and Nupr1 in treating sevoflurane-induced neurotoxicity.

## MATERIALS AND METHODS

### Ethics statement

The Zhejiang University Institutional review board of Animal studies approved the study protocol (ZJU2019-16340). All experimental procedures were performed in accordance with the Guidance Recommendations for Experimental Animal Care and Use of the Ministry of Science and Technology of the People’s Republic of China (2006) [20].

### Animals and anesthesia treatment

Seven-day-old male Sprague-Dawley rats (18–20 g) were obtained from the Laboratory Animal Center of Zhejiang University (2019000514485). Animals from the same litter were distributed equally between the experimental and control groups to generate littermate controls for each experimental condition. All animals were raised in standard animal cages and maintained under a 12 h light-dark cycle at 22 ± 1°C. After they were randomly divided between the sevoflurane and control groups (n=6 per group), rats were exposed to either 3.4% sevoflurane (44071, Maruishi, Japan) in air or air alone for six hours in an anesthesia agent evaporator chamber. Rat body weights were measured seven days after birth and eight weeks after sevoflurane or air exposure.

### Arterial blood pressure and arterial blood gas analysis

A single 100 μL blood sample was obtained from the left cardiac ventricle of each rat via quick arterial blood sampling using a 24 gauge catheter immediately before and after exposure to sevoflurane or air for arterial blood-gas analysis. Arterial oxygen partial pressure (PaO_2_), arterial carbon dioxide partial pressure (PaCO_2_), power of hydrogen (PH), and blood oxygen saturation (SaO_2_) were measured using a Nova Biomedical blood gas apparatus (ABL800; Radiometer, Copenhagen, Denmark). Mean arterial blood pressure (MAP) was measured using BIOPAC MP150 and AcqKnowledge software (Biopac Systems Inc., Goleta, CA).

### Cell culture and anesthesia treatment

HCN-2 human cortical neuron cells were purchased from ATCC (ATCC, Rockville, MD, USA) and cultured in Dulbecco’s Modified Eagle’s Medium (DMEM) supplemented with 15% fetal bovine serum (Invitrogen, Shanghai, China) in an incubator under 5% CO_2_ at 37°C. HCN-2 cell culture dishes were randomly divided between the experimental and control groups (n=6 each). The culture dishes were placed in an anesthesia induction chamber maintained at 37°C (RWD Life Science Co., Ltd., Shenzhen, China) and containing fresh gas (21% O_2_, 5% CO_2_, remainder N_2_) alone for the control group and with 3.4% sevoflurane for the experimental group (group S) for six hours. This sevoflurane concentration was steadily maintained throughout the experiments using a Capnomac gas monitor (Datex-Ohmeda, Helsinki, Finland).

### Transfection experiments and intracranial injections

Nupr1 siRNA and miR-325 mimic (Biomics Biotechnologies Co., Ltd, Nantong, China) were transfected into cells using Lipofectamine 2000 (Invitrogen, 11668-019) according to the manufacturer’s protocol. After transfection, cells were exposed to either 3.4% sevoflurane or fresh air for six hours; Nupr1, C/EBPβ, Bcl-2, Bax, and Caspase-3 expression were then evaluated via Western blots and quantitative real-time PCR. Neonatal rats were fixed in a stereotaxic apparatus (Kopf instruments) and given bilateral hippocampal injections of agomiR-325-3p or agomiR-NC (1.2 mm posterior to bregma, 1.5 mm lateral to the midline, and 1.8 mm below the dural surface) using a 28-gauge implantation cannula [21]. The rats were then exposed to sevoflurane one day after the injection.

### Western blot

Hippocampal tissue or HCN-2 cells were homogenized using RIPA lysis buffer (Boster Biotechnology, Wuhan, China) containing phenylmethanesulfonyl fluoride (Boster Biotechnology, Wuhan, China) for 15 min. Protein concentrations were then measured with a BCA kit (Beyotime Biotechnology, Haimen, China). Membranes were incubated overnight at 4°C with the following primary monoclonal antibodies: Anti-Nupr1 (ab6028), anti-Cleaved Caspase-3 (ab49822), anti-Bcl-2 (ab196495) (1: 1000 dilution; Abcam, UK), anti-C/EBPβ (2895), anti-IGFBP5 (10941), anti-Bax (2772), and anti-GAPDH (5174) (1:1000 dilution; Cell Signaling Technology, USA). Membranes were then incubated with horseradish peroxidase-labeled secondary mouse anti-rabbit (93702) or rabbit anti-mouse (5127) (1: 5000 dilution; Cell Signaling Technology, USA) antibodies for 2h. Super Signal West Pico Chemiluminescent Substrates (Pierce Biotechnology, Rockford, IL, USA) and GEL-PRO ANALYZER software (Bio-Rad Laboratories, Hercules, CA, USA) were used to visualize and quantify protein band absorbance.

### Quantitative real-time polymerase chain reaction (RT-qPCR)

Total RNA was extracted using RNAiso Plus (Takara Bio Inc, Shiga, Japan) and then reverse-transcribed to generate cDNA using the PrimeScript™ RT reagent Kit (Takara Bio Inc, Shiga, Japan) according to the manufacturer’s instructions. RT-qPCR was performed using the Step One Plus Real-Time PCR System (Thermo Fisher Scientific, Waltham, MA, USA). Nupr1 mRNA expression was analyzed using SYBR® Premix Ex Taq™ (Takara Bio Inc, Shiga, Japan). miR-325 expression was detected using the Mir-X miRNA First-Strand Synthesis Kit (Takara Bio Inc, Shiga, Japan). Expression data were analyzed according to the comparative Ct method and were normalized to β-actin or U6 levels. Primer sequences are listed below: Nupr1-F(rat): 5′-AGCCTGGCCCAAT CTTATGT-3′; Nupr1-R(rat): 5′-GGCCTAGGTCCTGCTTACAA-3′; NUPR1-F(human): 5′-GACTGAGTCTCTGAGGGGCTAC-3′ NUPR1-R (human): 5′-GTTGCTGCCACCCTGGAGGA-3′; β-actin-F: 5′-GGAGATTACTGCCCTGGCTCCTA-3′; β-actin-R: 5′-GACTCATCGTACTCCTGCTTGCTG-3′; rno-miR-325: 5′-TTTATTGAGCACCTCCTATCAA-3′; hsa-miR-325: 5′-CCTAGTAGGTGTCCAGTAAGTGT-3′.

### TUNEL assay

Brains were fixed through immersion in 5% formaldehyde in 0.1M phosphate-buffered saline (PBS; Gibco, 18912-014) at 4°C for 24h. TUNEL staining was performed on paraffin-embedded sections (5 μm) using a cell death detection kit (Roche Diagnostics GmbH, Mannheim, Germany) according to the manufacturer’s protocol; apoptotic cell nuclei turned brown in diaminobenzidine chromogenic liquid (K5007, DAKO, Denmark). Images were acquired using a DFC295 digital camera and a DM2500 bright-field light microscope with Leica Application Suite 4.1.0 software at 200× magnification; images were edited in Adobe Photoshop CS5 Version 12.1.32. Numbers of TUNEL-positive cells in each field were quantified using Image-Pro Plus software, and the apoptotic index was defined as the ratio of TUNEL-positive cells to total cells in each field.

### Apoptotic cell analysis

Cells were collected, cleared with cold PBS twice, resuspended in 1X Binding Buffer, and stained with Annexin V-FITC and PI with an Annexin V-FITC/PI apoptosis detection kit (Becton–Dickinson, San Jose, USA) according to the manufacturer’s protocol. The apoptosis ratio was measured using a BD FACS (Fluorescence Activated Cell Sorting) Accuri C6 (Becton-Dickinson, San Jose, USA). Cells are divided into three subsets using this sorting protocol: late apoptotic cells are double-stained with green and red fluorescence, early apoptotic cells show strong green fluorescence, and living cells exhibit very low-intensity background fluorescence.

### Dual-luciferase reporter gene assay

TargetScan was used to determine whether miR-325 directly targets the Nupr1 gene. Luciferase-reporters including wildtype and mutant 3′-UTRs were constructed (Promega, Beijing, China) and used in a dual-luciferase reporter gene assay kit (Promega) according to the manufacturer’s instructions.

### Novel object recognition test

The cognitive function of the rats was assessed eight weeks after sevoflurane exposure using the novel object recognition test. The test was carried out according to the published standard [22]. Rats were placed in the empty arena (without any objects) for 10 min 24 h before testing to familiarize them with the environment. During the introduction phase, two identical objects (X1 and X2, black metal cans) were placed in opposite corners approximately 15 cm from the walls of the field. Next, during the recognition phase, one of the previous objects was placed in the arena with a novel one (X = familiar black metal can, Y= novel green glass vase). The objects were weighted down with wet sand to prevent the rats from moving them. The position of the novel object during the recognition phase was randomly assigned for each rat. Exploration of an object was defined by touching, sniffing, or licking the object; leaning against the object and sniffing as well as sitting or standing on it were not considered exploration. Time spent on exploration and movement in the arena were recorded by two independent experimenters blind to the experimental design. Discrimination index (Di) was defined as time spent exploring the novel object relative to total exploration time for both objects and was calculated as follows: Di = time spent on novel object exploration x 100 / (time spent on novel object exploration + time spent on familiar object exploration).

### The open field test

Rat exploratory behavior and spontaneous motor activity were assessed 8 weeks after sevoflurane exposure in an open field test (OFT) [23]. Rats were gently placed in the OFT arena for 10 minutes for familiarization before the test began. They were then placed in a circular open field (100 cm in diameter) painted black and positioned 50 cm above the floor. The field was separated into 8 equal parts by thin white lines. The center of the field was illuminated by bright white light. Each rat was placed in the center of the arena and observed for 5 minutes before data recording began. For the next five minutes, the rat’s movement in the open-field chamber was automatically documented using Any-Maze animal tracking system software (Xinruan, Shanghai, China). Total distance traveled and amount of time spent in the center of the arena were also recorded by the software.

### Statistical analysis

Means ± standard deviation (SD) were used to describe all data, and SPSS 24.0 (SPSS, Inc., Chicago, IL, USA) was used for statistical analysis. Student’s t-tests were used to analyze numerical data; p < 0.05 was considered statistically significant.
